# Perturbation of Critical Prolines in *Gloeobacter violaceus* Ligand-gated Ion Channel (GLIC) Supports Conserved Gating Motions among Cys-loop Receptors*

**DOI:** 10.1074/jbc.M115.694372

**Published:** 2015-12-14

**Authors:** Matthew Rienzo, Angela R. Rocchi, Stephanie D. Threatt, Dennis A. Dougherty, Sarah C. R. Lummis

**Affiliations:** From the ‡ Division of Chemistry and Chemical Engineering, California Institute of Technology, Pasadena, California 91125 and; the § Department of Biochemistry, University of Cambridge, Tennis Court Road, Cambridge CB2 1GA, United Kingdom

**Keywords:** Cys-loop receptor, electrophysiology, non-standard mutagenesis, protein conformation, structure-function, Gloeobacter violaceus, proline analogs, non-canonical amino acid, nonsense suppression, mutagenesis

## Abstract

*Gloeobacter violaceus* ligand-gated ion channel (GLIC) has served as a valuable structural and functional model for the eukaryotic Cys-loop receptor superfamily. In Cys-loop and other receptors, we have previously demonstrated the crucial roles played by several conserved prolines. Here we explore the role of prolines in the gating transitions of GLIC. As conventional substitutions at some positions resulted in nonfunctional proteins, we used *in vivo* non-canonical amino acid mutagenesis to determine the specific structural requirements at these sites. Receptors were expressed heterologously in *Xenopus laevis* oocytes, and whole-cell electrophysiology was used to monitor channel activity. Pro-119 in the Cys-loop, Pro-198 and Pro-203 in the M1 helix, and Pro-299 in the M4 helix were sensitive to substitution, and distinct roles in receptor activity were revealed for each. In the context of the available structural data for GLIC, the behaviors of Pro-119, Pro-203, and Pro-299 mutants are consistent with earlier proline mutagenesis work. However, the Pro-198 site displays a unique phenotype that gives evidence of the importance of the region surrounding this residue for the correct functioning of GLIC.

## Introduction

The Cys-loop family of ligand-gated ion channels (LGICs)^2^ includes nicotinic acetylcholine receptors (nAChRs), serotonin receptors (5-HT_3_Rs), GABA_A_ receptors (GABA_A_Rs), and glycine receptors (GlyRs). These receptors are pentameric membrane-spanning proteins that mediate fast synaptic transmission throughout the nervous system (1, 2). They are members of a larger family, the pentameric ligand-gated ion channels (pLGICs), all of which share a pentameric structure and a similar global layout. Individual subunits contain a large extracellular N-terminal domain (ECD) and four transmembrane helices (M1–M4). Neurotransmitters generally bind in the ECD, at the interface between two adjacent subunits, triggering a conformational change that opens the pore, which is lined by the five M2 helices. The transduction of binding information to the pore region is not yet fully understood, partly because structural data for this family have lagged behind those for many other ion channels. However, relevant data are now becoming available, and recent publications describing the high-resolution structures of the first mammalian Cys-loop receptors (GABA_A_, 5-HT_3_, and glycine receptors) have contributed to our growing knowledge (3–5). Nevertheless, obtaining structural data on these proteins is challenging, and many in the field have turned to prokaryotic pLGICs (6, 7) as model systems due to their facile expression in heterologous systems and relative ease of crystallization. The most widely used pLGICs are those from *Erwinia chrysanthemi*, now known as *Dickeya dadantii* (*Erwinia* ligand-gated ion channel; ELIC) (8) and *Gloeobacter violaceus* (*Gloeobacter* ligand-gated ion channel; GLIC) (9–14). These prokaryotic receptors share the general structure of the Cys-loop receptors, but lack the large, variable intracellular domain as well as the cysteines in the eponymous Cys-loop. Electrophysiological characterization has revealed that these prokaryotic channels also share many functional attributes with the eukaryotic pLGICs, including conductance, cation selectivity, sensitivity to similar channel blockers, and comparable effects of pore mutations (7, 15). Over 40 structures of GLIC have been obtained under various crystallization conditions, and they are presumed to represent several different thermodynamic states of the receptor. This has allowed for evidence-based predictions of global gating transitions conserved among the pLGICs. GLIC thus offers a testing ground for corroboration of functional data from mutant receptors with hypothetical conformational changes.

Unusual among the pLGICs, GLIC is activated by a decrease in extracellular pH, rather than by a small-molecule ligand. The receptor is also unique in having proline residues that are not found in eukaryotic pLGICs in a number of locations. Proline is set apart from the other amino acids by its limited hydrogen-bonding capability, increased steric bulk at the backbone nitrogen, and greater propensity to exist in a cis conformation at the backbone peptide bond. These features are essential for function in many proteins, including some eukaryotic Cys-loop receptors (16–19). Understanding the roles of the proline residues in GLIC could allow for elucidation of structural transitions during activation and could help clarify whether GLIC is an appropriate model system to understand structure-function relationships in vertebrate Cys-loop receptors. Here we have substituted each of the proline residues in GLIC with alanine to identify any sensitive sites. Because conventional mutagenesis at some positions appears to ablate GLIC expression, trafficking, and/or function, we have also implemented the subtler probe of non-canonical amino acid mutagenesis, which permits small systematic perturbations to steric and electrostatic interactions. We identify several trends that agree with those previously observed in corresponding sites of the eukaryotic receptors (17–19), as well as several unusual effects that reveal regions important for the activation process.

## Experimental Procedures

### 

#### 

##### Molecular Biology

The cDNA for GLIC was in the pGEMhe plasmid. Site-directed mutagenesis was performed using the Stratagene QuikChange protocol to generate the appropriate codon. For non-canonical amino acid mutants and conventional mutants generated by nonsense suppression, the site of interest was mutated to the TAG stop codon. Plasmids were linearized with the SbfI restriction enzyme, and receptor mRNA was then prepared by *in vitro* runoff transcription using the Ambion T7 mMESSAGE mMACHINE kit.

Hydroxy or amino acid-dinucleotide conjugates were enzymatically ligated to truncated 74-mer THG73 tRNA as described previously (20, 21). The 74-mer tRNA was prepared using the Ambion MEGAshortscript T7 kit by transcription from a DNA oligonucleotide template modified to enhance RNA transcript homogeneity, as described in the literature (22). Crude tRNA amino acid or tRNA-hydroxy acid product was used without desalting, and the product was confirmed by matrix-assisted laser desorption ionization time-of-flight mass spectrometry on a 3-hydroxypicolinic acid matrix. Deprotection of the nitroveratryloxycarbonyl group on the tRNA amino acids was carried out by photolysis for 5 min on a 300-watt high-pressure mercury arc lamp with WG-335 and UG-11 filters immediately prior to injection.

##### Oocyte Preparation and RNA Injection

Stage V–VI oocytes of *Xenopus laevis* were harvested and injected with RNAs as described previously (21). For nonsense suppression experiments, each cell was injected with 50–100 ng each of receptor mRNA and appropriate tRNA ∼48 h before recording. Mutants yielding small responses required 72 h of incubation, with a second injection of mRNA and tRNA 48 h before recording.

For wild type experiments and conventional mutants, each cell received a single injection of 1–25 ng of receptor mRNA ∼24 h before recording. Injection volumes for each injection session were 50–100 nl/cell.

As a negative control for suppression experiments at each site, unacylated full-length tRNA was co-injected with mRNA in the same manner as charged tRNA. These experiments yielded negligible responses for all sites. Wild type recovery conditions (injecting tRNA charged with the appropriate amino acid to regenerate a wild type channel via nonsense suppression at a TAG stop codon) were injected alongside mutant nonsense suppression conditions as a positive control.

##### Electrophysiology

Oocyte recordings were made in two-electrode voltage clamp mode using the OpusXpress 6000A (Axon Instruments). Oocyte equilibration and washes were performed with calcium-free ND96 (96 mm NaCl, 2 mm KCl, 1 mm MgCl_2_, 5 mm HEPES) adjusted to pH 8 with 1 n NaOH. The pH values of buffers for concentration-response curves were adjusted accordingly with NaOH or HCl, and buffers with pH below 6.8 contained 5 mm MES in place of HEPES. Initial holding potential was −60 mV. Data were sampled at 125 Hz and filtered at 50 Hz. Oocytes were equilibrated for 30 s at 1 ml/min before each pH application. The pH buffer applications lasted for 15 s at 4 ml/min, followed by a 15-s wait period to allow the cells to attain a peak current. Cells were then washed for 40 s at 3 ml/min before the following equilibration. Concentration-response data were obtained for nine buffer pHs, for a minimum of two cell batches, and for a minimum of seven cells total (or 3–4 cells from 1–3 batches for alanine scanning). The concentration-response relations for each cell were fitted to the Hill equation, *I*_norm_ = 1/(1 + (EC_50_/*A*))*^n^*^H^, where *I*_norm_ is the normalized current peak at [H_3_O^+^] = *A*, EC_50_ is the value of [H_3_O^+^] that elicits a half-maximum response, and *n*_H_ is the Hill coefficient. The values for each cell were then averaged to give the reported values, where pH_50_ = −log(EC_50_). At pH < 4, uninjected cells gave current responses that were occasionally up to ∼200 nA. As such, data were only reported from cells that gave responses above pH 4, or cells that gave larger, more robust currents below pH 4, and other cells were considered non-responsive (NR).

## Results

### 

#### 

##### Conventional Mutagenesis Identifies Four Sensitive Proline Sites

**FIGURE 1.**
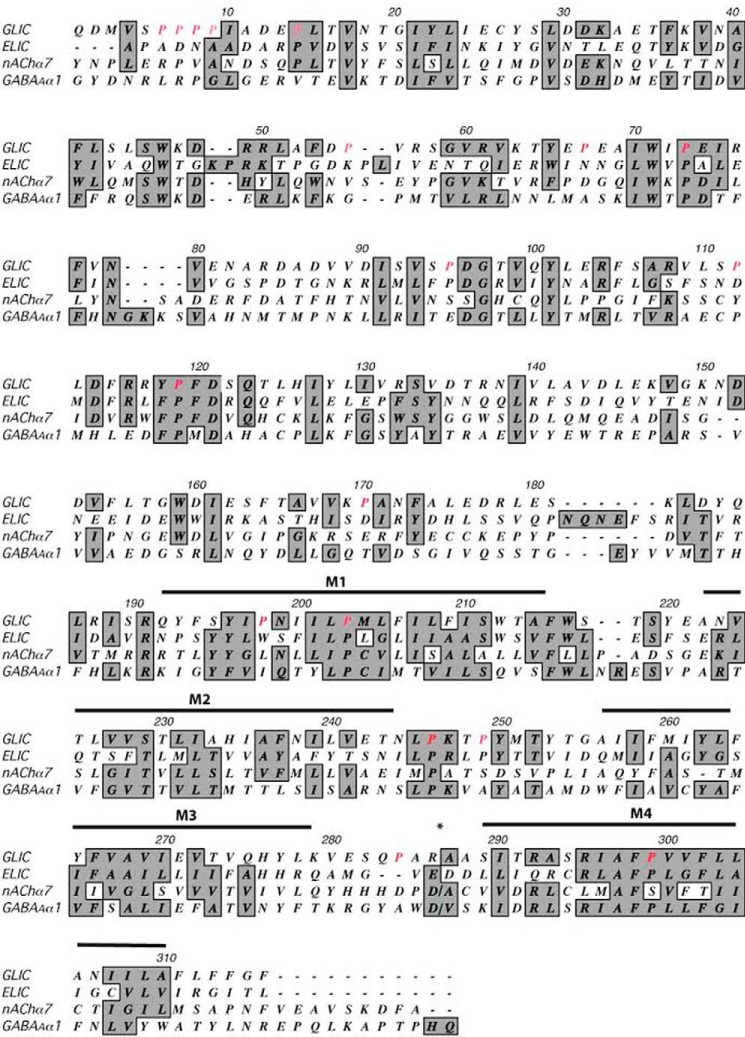
**Sequence alignment of GLIC, ELIC, and two example eukaryotic pLGIC subunits: nAChR α7 and GABA_A_ R α1.** Prolines examined in this study are shown in *red. Shaded residues* are conserved or similar in side-chain character among sequences. The M3/M4 loop of the eukaryotic receptors has been removed at *.

**FIGURE 2.**
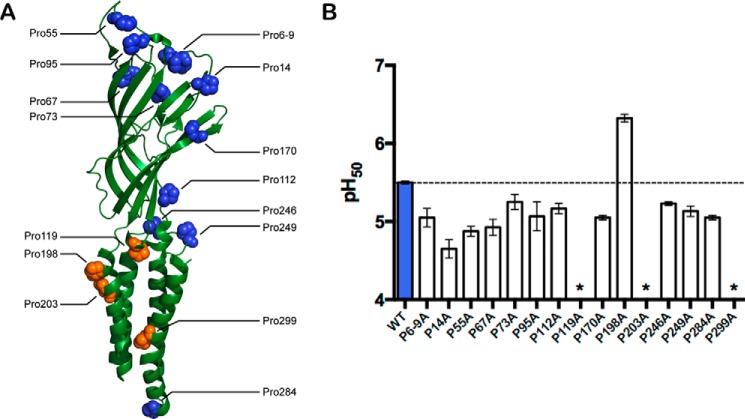
**Proline sites examined in this study.** *A*, GLIC subunit structure, indicating proline residues (PDB: 3EHZ). *B*, conventional mutagenesis of GLIC Pro residues. Substitution with Ala mostly had little or no effect on pH_50_ values, although P119A and P203A were nonfunctional, and P198A had increased sensitivity. *P6-9A* indicates simultaneous substitution of prolines at sites 6, 7, 8, and 9 with Ala; individual Ala substitutions at these sites yielded similar pH_50_ values (not shown). Data shown are mean ± S.E., *n* = 3–4. *, pH-induced responses were comparable with uninjected cells. Typical maximal responses for WT and all Ala-containing mutants were 20–40 μA, except for P14A, where maximal responses were 2–5 μA.

GLIC contains 18 proline residues, 12 of which are also present in related receptors (Fig. 1). Upon mutation to alanine, 14 sites gave functional receptors with only minor decreases in pH_50_ (Table 1; Fig. 2), suggesting that these residues are not critical for channel gating. However, conventional mutations at 3 conserved sites (Pro-119, Pro-203, and Pro-299) ablated acid-induced currents, and mutation at Pro-198, unique to GLIC, resulted in an increased sensitivity to protons (*i.e.* higher pH_50_). We therefore proceeded to examine these four sites more closely with the aid of non-canonical amino acid analogs.

**TABLE 1:** **pH_50_ values and Hill coefficients of unnatural proline mutations examined in this study** Data shown are mean ± S.E. NR indicates that acid-induced responses were comparable to those recorded from uninjected oocytes. Ala, Leu, and Gly mutants were generated by conventional mutagenesis. All other mutants were generated via nonsense suppression.

Site	Mutation	pH_50_	Hill slope
Pro-119	Ala	NR	NR
	Leu	NR	NR
	Gly	NR	NR
	Pro	5.40 ± 0.06	1.6 ± 0.1
	Aah	NR	NR
	Lah	NR	NR
	*N*-MeAla	NR	NR
	Aze	NR	NR
	Pip	6.34 ± 0.02	2.1 ± 0.1
	Mor	6.35 ± 0.02	3.1 ± 0.1
	P3m*^a^*	6.47 ± 0.01	1.5 ± 0.1
	Dhp	NR	NR
	Flp	5.58 ± 0.07	1.7 ± 0.2
	flp	NR	NR

Pro-198	Ala	6.70 ± 0.03	0.7 ± 0.1
	Leu	6.37 ± 0.03	2.4 ± 0.2
	Gly	6.48 ± 0.02	2.4 ± 0.2
	Pro	5.64 ± 0.02	1.6 ± 0.1
	Aah	5.29 ± 0.02	1.4 ± 0.1
	Lah	5.33 ± 0.05	1.4 ± 0.1
	*N*-MeAla	5.33 ± 0.05	1.2 ± 0.1
	Aze	5.88 ± 0.04	1.7 ± 0.1
	Pip	5.53 ± 0.03	1.2 ± 0.1
	Mor	5.93 ± 0.02	1.7 ± 0.1
	P3m	5.76 ± 0.06	1.2 ± 0.1
	Dhp	5.42 ± 0.03	1.3 ± 0.1
	Flp	6.10 ± 0.05	1.5 ± 0.1
	flp*^a^*	6.52 ± 0.02	1.9 ± 0.2

Pro-203	Ala	NR	NR
	Leu	NR	NR
	Gly	NR	NR
	Pro	5.61 ± 0.01	1.9 ± 0.1
	Aah	NR	NR
	Lah	NR	NR
	*N*-MeAla	5.24 ± 0.03	1.7 ± 0.1
	Aze	NR	NR
	Pip	NR	NR
	Mor	4.73 ± 0.02	2.0 ± 0.1
	P3m*^b^*	<4.5	
	Dhp	5.12 ± 0.04	1.4 ± 0.1
	Flp	NR	
	flp	5.63 ± 0.02	2.0 ± 0.1

Pro-299	Ala	NR	
	Leu	NR	
	Gly	NR	
	Pro	5.52 ± 0.02	1.6 ± 0.1
	Aah	NR	
	Lah	5.44 ± 0.02	1.3 ± 0.1
	*N*-MeAla	5.53 ± 0.05	1.5 ± 0.1
	Aze	NR	
	Pip	5.77 ± 0.03	1.6 ± 0.1
	Mor	5.75 ± 0.02	1.6 ± 0.1
	P3m	5.60 ± 0.03	1.4 ± 0.1
	Dhp	5.41 ± 0.04	1.4 ± 0.1
	Flp	6.01 ± 0.02	2.2 ± 0.1
	flp	5.91 ± 0.01	1.6 ± 0.1

*^a^* A biphasic curve was observed with a low-sensitivity component having pH_50_ <4.5.

*^b^* Currents were observed, but pH_50_ was too low to measure (<4.5), due to nonspecific acid-induced currents.

##### The Cys-loop Tyr/Phe-Pro Motif Shows Sensitivity to Pro Cis Bias and Hydrophobicity

**FIGURE 3.**
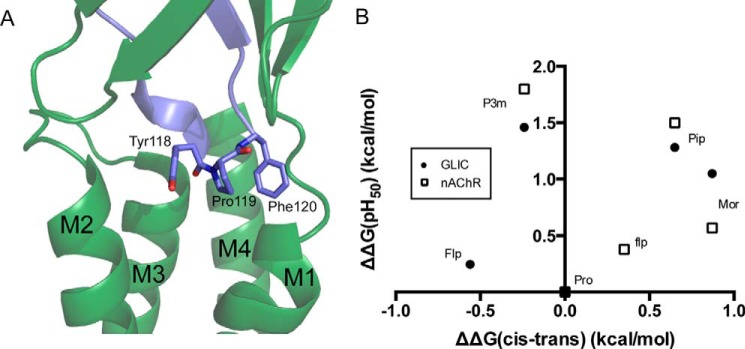
**The GLIC YPF motif.** *A*, the ECD/TM interface in GLIC, highlighting the YPF motif in the cis conformation (PDB: 3EAM). *B*, relationship between mean pH_50_ values and cis-trans preferences for Pro analogs at Pro-119 in GLIC (*filled circles*), when compared with the analogous position (Pro-136) in the muscle-type nAChR (*open squares*) (17). Typical maximal currents for functional mutants generated by nonsense suppression were 0.5–2 μA, with P119Pip giving responses as high as 10 μA.

The Cys-loop, a disulfide-containing region at the ECD/TM interface, is a unifying trait of the eukaryotic pLGICs and has long been thought to contribute to transduction of ligand binding information to the transmembrane domain (16, 23, 24) (Fig. 3). Although GLIC lacks the 2 cysteine residues usually found in this loop, the region is structurally similar to the vertebrate receptors. The first proline we identified as essential for function, Pro-119, is located in this loop, and is a highly conserved feature of the Cys-loop of the eukaryotic pLGICs. Notably, this proline is generally flanked by 2 aromatic residues. It is well established that aromatic residues immediately preceding proline sites preferentially stabilize the cis backbone conformation of the proline (17, 25). Indeed, the highest resolution x-ray crystal structure of GLIC shows this proline in the cis configuration (26). In other pLGIC structures, including one of GLIC, this proline is in a trans conformation, (4, 8–10), but this is a subtle distinction that may be beyond the resolution of some structures. At present, we cannot rule out the possibility that both conformers can exist in functional pLGICs, bringing up the intriguing possibility that they may be linked to different receptor states.

**FIGURE 4.**
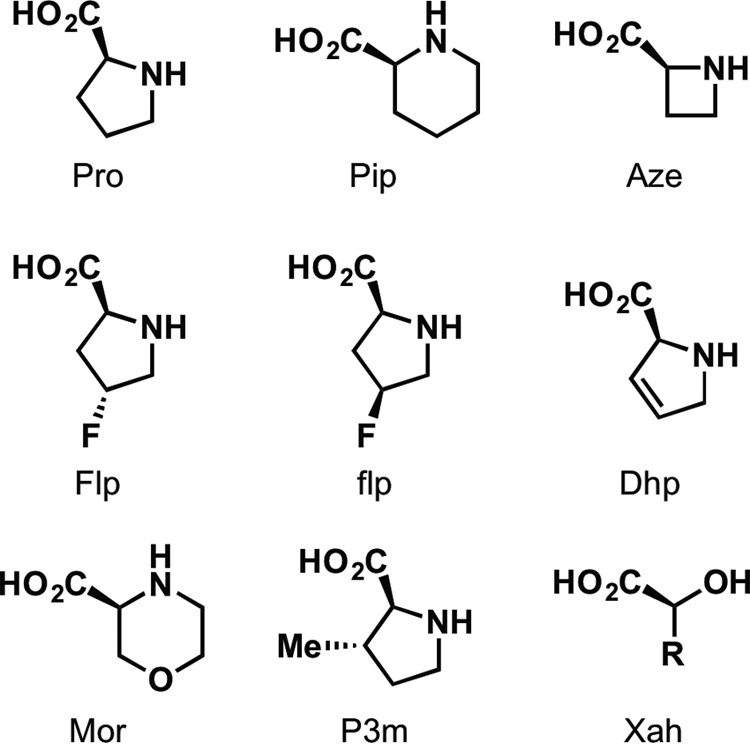
**Structures of amino acid analogs used in this study.**

Previous studies from our group have investigated the potential of various proline residues in membrane receptors to undergo cis-trans isomerization as a step in the process of channel gating, including a site homologous to Pro-119 in a nAChR (16, 17). To determine whether such an isomerization at Pro-119 might be involved in GLIC gating, we performed mutagenesis with a number of canonical and non-canonical amino acids. Our data revealed this proline to be highly sensitive, yielding no functional receptors when substituted with Ala, Leu, or Gly (Table 1). Many prolines serve to disrupt secondary structure because their backbone nitrogen lacks the hydrogen bond-donating site present in the other amino acids. To see whether this condition would be sufficient to allow the channel to function, we introduced Aah and Lah, the α-hydroxy acid analogs of Ala and Leu (Fig. 4), which also lack a backbone hydrogen bond donor. Such mutations at Pro sites often produce fully functional receptors. However, at Pro-119 of GLIC, these analogs resulted in no functional responses. This is a phenotype that would be expected if interconversion between cis and trans forms were required for channel activation because all of these substitutions would disfavor isomerization, but it is also consistent with a cis orientation being essential for function. Interestingly, two analogs with increased intrinsic cis biases, the six-membered rings Pip and Mor, engendered an increase in sensitivity to acid. This effect was similar to that which was observed in the nAChR (Table 1; Fig. 3*B*). We considered an additional residue, Aze, which is also expected to have an increased cis bias, but we were unable to observe receptor function when we attempted to incorporate Aze.

In the case of the nAChR, receptor function markedly favored hydrophobicity at both the proline and the preceding aromatic residue (17). Introduction of methyl groups on either side chain resulted in lower EC_50_ values than were expected based on the proline cis preference, whereas the more polar Mor and flp gave the opposite effect. Although a similar trend was observed with P3m and Flp at the GLIC site, Mor showed nearly the same pH_50_ as Pip. The aromatic residue preceding Pro-119 in GLIC is tyrosine, but it is phenylalanine in the nAChR and other Cys-loop receptors. We therefore repeated the experiment in combination with the Y118F mutation to determine whether this difference in polarity could account for the altered hydrophobicity effect. In the Y118F background, the Mor- and Pip-containing mutants still yielded very similar pH_50_ values (Table 2), indicating that other factors intrinsic to GLIC must be responsible for the observed phenotype.

**TABLE 2:**
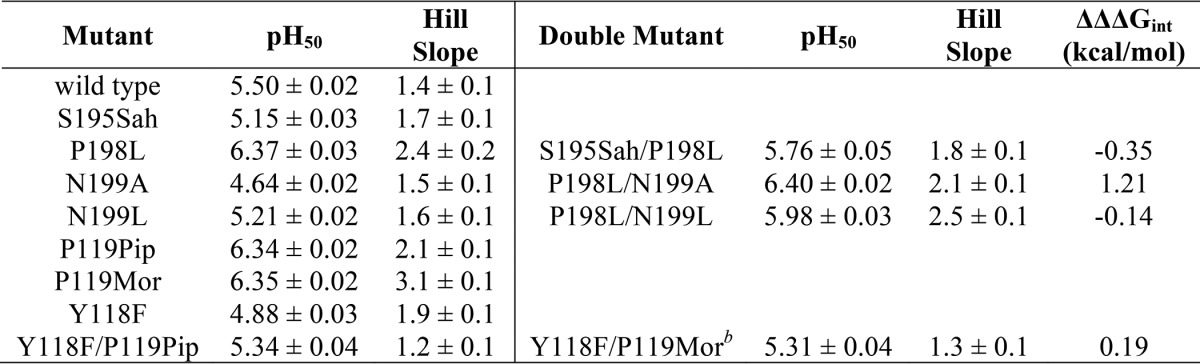
**Mutant cycle analyses for interactions at the extracellular terminus of M1*^a^***

*^a^* ΔΔΔ*G*_interaction_ was calculated from the formula ΔΔΔ*G*_interaction_ = −*RT*ln((EC_50_WT · EC_50_Mut1,2)/(EC_50_Mut1 · EC_50_Mut2)) where EC_50_ = 10^−pH_50_^.

*^b^* Interaction energy was calculated using P119Pip, rather than wild type, as a reference point.

Flp and flp maintain roughly equivalent steric and polarity contributions, mainly varying in the direction of pucker in the cyclic side chain (27). This effect can in turn influence the cis-trans equilibrium of the proline, as has been previously shown in a model peptide containing a Tyr-Pro motif; Flp, the trans stereoisomer, favors an *exo* pucker and has a strong trans bias (relative to Pro, ΔΔ*G* = −0.56), whereas the epimer flp favors an *endo* pucker and a relative cis bias (ΔΔ*G* = 0.35) (28). In contrast to the nAChR, we observed currents only from receptors containing Flp, the trans stereoisomer, and not from flp. Based on the structures showing either cis or trans conformations at this site, the fluoro group on Flp is expected to point into the Tyr side chain, whereas the fluoro group on flp should point in the opposite direction, toward the subsequent Phe. This suggests a high degree of sensitivity not only to the polarity of the side chain, but also to the particular orientation and localization of the dipole.

A number of the mutations at Pro-119 resulted in negligible currents, and so disruption of surface expression must also be considered as a possibility, especially for the more dramatic conventional mutations. However, as several proline analogs that introduce moderate steric perturbations are readily tolerated, it seems most likely that the lack of current seen in other cases is due to an effect on gating transitions.

##### A Helical Distortion in M1 Is Involved in Activation

The second conserved proline that was intolerant to alanine substitution, Pro-203, is located in the M1 helix. A similar proline is present in all pLGICs and has been shown to be essential for receptor activity in nAChRs and 5-HT_3_ receptors (18, 19). In the Cys-loop receptors, this proline displays a distinctive phenotype: conventional mutants give a nonfunctional channel, but incorporation of α-hydroxy residues, regardless of side chain, yields robustly expressing, ligand-activated receptors. This establishes that of the several unique features of a proline residue, lack of a backbone hydrogen bond donor is the essential requirement here.

**FIGURE 5.**
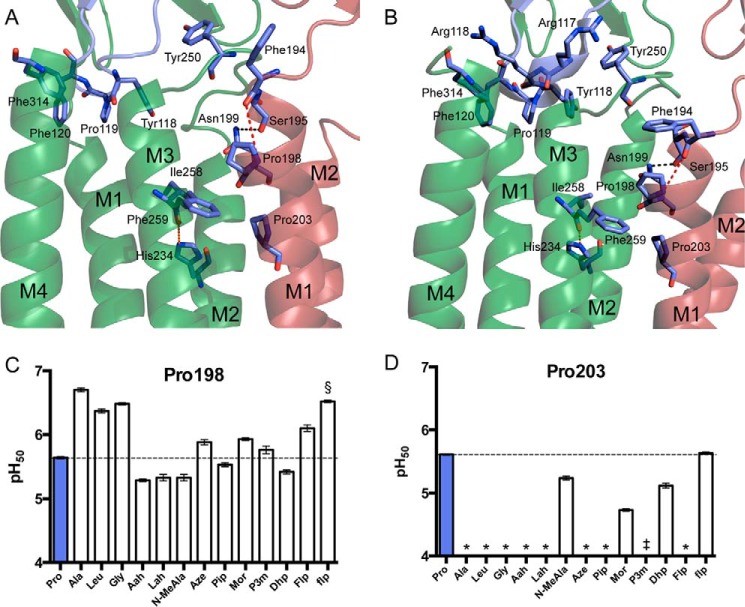
**The GLIC M1 prolines.** *A* and *B*, Pro-198 and Pro-203 in the top of the M1 helix at pH 7 (PDB: 4NPQ) (*A*) and pH 4 (PDB: 3EHZ) (*B*). Shown are a hydrogen bond between Asn-199 and the main-chain carbonyl of Ser-195 (*black*), the disrupted hydrogen bond hypothesized to be restored by conventional mutagenesis at Pro-198 (*red*), and the interhelix hydrogen bond between His-234 and the Ile-258 carbonyl (*green*). *C* and *D*, pH_50_ values for GLIC Pro-198 (*C*) and Pro-203 (*D*) mutants. Data shown are mean ± S.E., with *n* = 8–20. Conventional mutants at Pro-198 gave currents comparable with wild type GLIC. Typical maximal currents from functional mutants generated by nonsense suppression were 0.5–3 μA, with P198Lah giving responses as high as 20 μA. *, pH-induced responses comparable with uninjected cells; §, a biphasic curve was observed with a low-sensitivity component having pH_50_ < 4.5; ‡, currents were observed, but pH_50_ was too low to measure (< 4.5) due to nonspecific acid-induced currents.

In GLIC, conventional mutants at Pro-203 similarly gave negligible current responses. Surface expression of the P203L mutant was verified by Western blotting of stripped oocyte membranes (data not shown), indicating that mutations at this site are capable of rendering the receptor nonfunctional, rather than interrupting assembly or trafficking. However, we were unable to rescue function by ablation of the backbone NH; α-hydroxy analogs gave nonfunctional channels. In fact, Pro-203 proved to be extremely sensitive to substitution (Table 1; Fig. 5*D*). Even the very close proline analogs Pip and Aze did not produce functional receptors. In contrast, *N*-methylalanine (*N*-MeAla), which ablates the backbone NH but also lacks proline's cyclic side chain, did result in functional receptors. The substituted proline analogs P3m and flp, as well as the unsaturated Dhp, also gave functional channels.

This difference in phenotype between GLIC and the eukaryotic Cys-loop receptors could be related to a unique feature of the GLIC M1 helix: a second proline, Pro-198, located a single helix turn away from Pro-203. The presence of these two juxtaposed prolines results in a more dramatic helix deformation than is normally observed in structures of pLGIC M1 helices (Fig. 5, *A* and *B*). Pro-198 mutants generally retained function (Fig. 5*C*). Most perturbations at Pro-198 that preserved proline's lack of a backbone hydrogen bond donor displayed a pH_50_ within 0.3 units of the wild type receptor. However, mutations to the canonical amino acids Gly, Ala, or Leu resulted in a gain of function (activation at lower acid concentrations). These mutants could potentially allow formation of a backbone hydrogen bond between the introduced NH and the carbonyl of Phe-194, perhaps stabilizing the M1 helix and favorably influencing the open conformation. To test this hypothesis, we weakened the hydrogen-bonding ability of the Phe-194 carbonyl via incorporation of an α-hydroxy acid at the adjacent residue, Ser-195. The resulting mutant P198L/S195Sah does indeed show a more basic pH_50_ value than that of the single mutant P198L (Table 2). However, examination of the S195Sah mutation alone reveals that the effects of the two mutations are nearly additive, suggesting that this hydrogen-bonding interaction may not be responsible for the observed gain of function in the conventional Pro-198 mutants.

In another effort to examine the importance of hydrogen bonding in this region, we mutated Asn-199, the side chain of which forms a strong hydrogen bond with the Ser-195 main-chain carbonyl in the crystal structure (Fig. 5, *A* and *B*). Although the isosteric N199L mutant produced only a minor effect, removal of the bulky side chain in N199A dramatically reduced pH_50_, indicating important steric requirements in the intersubunit region immediately surrounding Pro-198. Furthermore, introduction of the N199A mutation in the presence of P198L results in no change in pH_50_ when compared with the proline mutation alone. This suggests that the effects of a conventional mutation at Pro-198 obviate the need for steric bulk at the side chain of Asn-199, pointing to a modified intersubunit interaction as the origin of the gain of function.

##### The M4 Pro Residue

**FIGURE 6.**
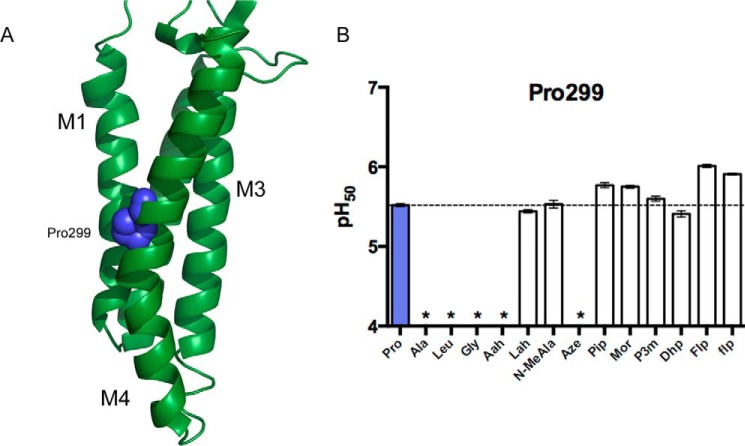
**GLIC Pro-299.** *A*, The GLIC TM domain, highlighting Pro-299 (PDB: 3EHZ). *B*, pH_50_ values for GLIC Pro-299 mutants. Data shown are mean ± S.E., with *n* = 7–20. Typical maximal currents from functional mutants generated by nonsense suppression were 0.5–5 μA. *, pH-induced responses comparable with uninjected cells.

Recent data have indicated that the M4 helix is a highly important region of pLGICs that is involved in allosteric modulation of receptor function via interaction with lipids, the adjacent M1 and M3 helices, and the extracellular domain (29, 30). The final sensitive residue identified in GLIC, Pro-299, is located in the middle of the M4 helix (Fig. 6*A*) and has recently been identified as functionally important in an alanine screen of the M4 helix (31). Although a homologous proline does not appear in the nAChRs or 5-HT_3_Rs, it is conserved in the GABA_A_Rs, GlyRs, and ELIC. In each of the available structures containing a proline in this position, a clear and dramatic helix kink is observed. Conventional mutants at this site resulted in nonfunctional receptors. In contrast, with the exception of Aah and Aze, most analogs lacking a backbone NH rescued function, giving close to wild type characteristics (Fig. 6*B*). Similar to the behavior typically observed for the M1 proline of Cys-loop receptors (but not in the case of GLIC), this pattern indicates that disruption of the helix by deletion of a hydrogen bond is sufficient for maintaining the observed kink, and therefore, channel function.

## Discussion

Previous studies concerning the functions of conserved prolines in transmembrane receptors (16–19, 32) have given rise to several commonly observed phenotypes: 1) those for which cis bias influences activity, 2) those for which ablation of a backbone hydrogen bond suffices to produce near wild type function, and 3) those for which steric bulk at the amine is necessary. Of the prolines examined above, Pro-119 conforms to the first group, Pro-299 conforms to the second, and Pro-203 conforms to the last. Examination of the available GLIC structural data also reveals that the conformational effects conferred by these residues are consistent with those of previously studied prolines with the same phenotypes: Pro-119 is located in a potentially flexible loop; Pro-299 forms a kink in M4 due to simple ablation of a hydrogen bond; and Pro-203 exists below a prominent bulge and displays steric requirements we have previously reported for a conserved bulge-inducing proline in the D2 dopamine receptor (32).

Although structural information from GLIC in a number of states has been obtained, it remains to be determined whether the gating motions observed in GLIC mirror the global conformational changes that govern activation of the eukaryotic Cys-loop receptors. Despite the lack of the disulfide in the loop containing Pro-119, both the structure of the loop and the effects of cis bias and hydrophobicity at Pro-119 closely resemble what we have observed at the analogous site in the nAChR. This supports the notion that during activation, any reorganization involving the Tyr/Phe-Pro motif is likely conserved, at least between nAChRs and GLIC. In the open GLIC structure (Protein Data Bank (PDB) 3EHZ), this loop directly contacts the M2–M3 loop via hydrophobic interactions and a cation-π interaction between Arg-116 and Tyr-250 (Fig. 5*B*), which suggests that the conformation of the “Cys-loop” could be coupled to the gating motions of M2. As pH_50_ data for Aze could not be obtained, it is difficult to conclude whether a cis conformation at this site is required for channel function, or whether a cis-trans isomerization is linked to gating transitions. However, considering the failure of this site to tolerate residues without an appreciable cis contributor and the gain of function observed upon incorporation of several cis-biased analogs, one of these possibilities is likely.

The M1 proline corresponding to Pro-203 in GLIC is conserved throughout the pLGIC superfamily, and in two Cys-loop receptors, we find a common phenotype: any residue that lacks a backbone NH produces an essentially wild type receptor (18, 19). In GLIC, however, this proline faces somewhat more stringent requirements: the necessity for substitution at the backbone nitrogen of Pro-203 suggests that the pronounced bulge in M1 observed in GLIC crystal structures must be enforced for activation to occur. Examination of the closed (pH 7) and open (pH 4) crystal structures (13, 14) has identified a difference in the interface between the extracellular end of M1 and the M2–M3 loop of the adjacent subunit. In the open structures, the pre-M1 region is tucked under the adjacent M2–M3 loop, with the nonconserved Pro-249 sandwiched between Phe-194 and Gln-192. In contrast, the closed structure shows Phe-194 pointed away from M1 into lipid, whereas the M2–M3 loop has shifted so that Pro-249 has moved past Gln-192. It is likely that this shifting occurs concurrently with or prior to the bend observed in M2 in the open structures. Substitution of Pro-203 with analogs bearing a hydrogen bond donor may result in a more ordered helical structure at the top of M1, eliminating any flexibility that is enabled by the presence of the bulge in the wild type receptor. This could prevent the motion of the M2–M3 loop, which may be required to initiate the bending of M2 observed in the open state.

Alternatively, or in addition, perturbation of Pro-203 could interfere with the correct alignment of a hydrogen bond that we have previously shown to be crucial for stabilization of the open state (33). In that work, we showed that a backbone amide-to-ester substitution at Phe-259 attenuates the hydrogen bond-accepting ability of the Ile-258 carbonyl and that tight steric complementarity makes this entire region highly intolerant to modification. The side chain of Phe-259 is in van der Waals contact with both Pro-198 and Pro-203; thus the bulge created by Pro-203 allows a direct interaction with the M3 helix of the adjacent subunit, which may be essential for function.

Interestingly, the nearby Pro-198 does not seem to be required to maintain this disruption in M1 and does not suffice to rescue function when Pro-203 is substituted. The distinct and robust increase in proton sensitivity conferred by conventional mutation of Pro-198 lies in stark contrast with the extreme sensitivity of Pro-203. Because mutations that introduce a backbone NH at Pro-198 cause a gain of function, whereas those that maintain proline's lack of a hydrogen bond donor produce receptors with wild type-like properties, it is tempting to argue that the effect we observe is simply due to an increased ordering of the N terminus of M1. However, the nearly additive effects we observed in the mutant cycle analysis with S195Sah make it difficult to conclude whether this is the primary cause of the shift in activity. The P198L and N199A mutations, on the other hand, are clearly non-independent. The importance of stabilizing interactions in the intersubunit region near the top of M1 has previously been established by structural studies on ethanol potentiation of GLIC mutants (34). Restructuring of this region caused by the conventional Pro-198 mutations could potentially allow for formation of comparable interactions in the absence of ethanol, leading to the observed pH sensitivity increase.

If the available structures accurately represent the open and resting conformations of GLIC, it does not appear that any major changes occur in the conformation or position of the M4 helix between these states. However, recent mutagenesis experiments examining the GLIC M4 helix have indicated that interactions between M4 and M1, M3, and the ECD are critical for receptor function (31). In that study, mutations of two or more aromatic residues in this region at the interface with M1 or M3 resulted in nonfunctional receptors, and so it is most likely that elimination of the M4 kink simply prevents the helix from adopting a conformation where these crucial interactions can be maintained. It is also possible that the correct structure of the GLIC M4 is essential for folding and trafficking of receptors as proper folding of M4 is required for cell surface trafficking in some other pLGICs. Various nAChR subunits, for example, have endoplasmic reticulum retention motifs in the pre-M1 region, which are masked when M4 binds to M1/M3, permitting cell surface expression (35). However, these residues are not well conserved in GLIC, and it is unclear whether a similar mechanism is operative.

In conclusion, we have identified several highly sensitive proline residues in GLIC. We show that the gating energetics for GLIC appear to depend significantly on the structure of the YPF motif of the Cys-loop, similar to what has previously been seen in eukaryotic nAChRs (17). We find that the highly conserved M1 proline maintains its critical role, albeit with a higher level of stringency than in the 5-HT_3_Rs or nAChRs. Both these results help to validate GLIC as a relevant homolog in the study of the gating process of the Cys-loop receptors. The M1 and M4 sites obey previously observed phenotypes for bulge- and kink-inducing prolines, respectively, reinforcing our devised classification strategy (32). Finally, we highlight the importance of stabilizing interactions in the intersubunit space adjoining the extracellular terminus of M1 based on unusual effects of substitutions to the Pro-198 site. Further studies will be required to delineate the structural consequences of mutations at this position, and may illuminate poorly understood aspects of the activation process.

## Author Contributions

M. R., S. C. R. L., and D. A. D. designed the study and wrote the paper. M. R., S. D. T., S. C. R. L., and A. R. R. prepared the mutant mRNA. M. R. prepared the aminoacyl tRNAs. M. R., S. D. T., and S. C. R. L. injected oocytes and performed electrophysiology experiments. All authors analyzed the results and approved the final version of the manuscript.

## Footnotes

2 The abbreviations used are:

LGIC: ligand-gated ion channel
pLGIC: pentameric LGIC
GLIC: *Gloeobacter* ligand-gated ion channel
nAChR: nicotinic acetylcholine receptor
ECD: extracellular domain
TM: transmembrane
*N*-MeAla: *N*-methylalanine
NR: non-responsive
Aze: azetidine-2-carboxylic acid
Aah: lactic acid
Lah: 2-hydroxyisocaproic acid
Pip: pipecolic acid
Mor: morpholine-3-carboxylic acid
P3m: 3-methylproline
Dhp: 3,4-dehydroproline
Flp: trans-3-fluoroproline
flp: cis-3-fluoroproline.
